# DNase I‐Mediated Chemotactic Nanoparticles for NETs Targeting and Microenvironment Remodeling Treatment of Acute Ischemic Stroke

**DOI:** 10.1002/advs.202503689

**Published:** 2025-06-19

**Authors:** Tongyu Zhang, Peixin Liu, Wenru Shen, Chao Li, Zhenhao Zhao, Yuxing Wu, Tao Sun, Chen Jiang

**Affiliations:** ^1^ Department of Pharmaceutics School of Pharmaceutical Sciences Fudan University Shanghai 201203 China; ^2^ Key Laboratory of Smart Drug Delivery Ministry of Education State Key Laboratory of Brain Function and Disorders Shanghai 201203 China

**Keywords:** acute ischemic stroke, nanomedicine, neurovascular unit, neutrophil extracellular traps, targeted delivery

## Abstract

The recruitment and formation of neutrophil extracellular traps (NETs) by neutrophils play an important role in reperfusion injury in ischemic stroke. Current nanosystem‐based therapeutic strategies are mainly confined within the blood‐brain barrier (BBB), ignoring the constant intrusion from external challenges. Here, considering the unique vascular localization of NETs, a DNase I‐mediated NETs‐targeting nanoparticle is developed to integrate the catalytic and chemotactic functions of DNase I and achieve the synergistic regulation of the internal and external microenvironment of the neurovascular unit (NVU). DNase I navigates the nanoparticles to the lesion, enabling the accumulation in the brain through damaged BBB. The removal of intravascular NETs mitigates the ongoing destruction of the endothelium and reduces the recruitment of immunothrombosis. The main nanoparticles with dual antioxidant activity rescue neuronal apoptosis by scavenging reactive oxygen species (ROS) and protecting mitochondria. Reduced infarct size and remodeling of microenvironment homeostasis shown in the middle cerebral artery occlusion/reperfusion (MCAO) mouse model. This strategy provides new insights into the vascular side treatment of ischemic stroke. Targeting mediated by enzyme chemotaxis is first validated and showed the potential of a universal chemotactic targeted delivery strategy.

## Graphical abstract

The neutrophil extracellular traps (NETs) play an important role in reperfusion injury in ischemic stroke. Here, considering the unique vascular localization of NETs, a DNase I‐mediated NETs‐targeting nanoparticle is developed to integrate the catalytic and chemotactic functions of DNase I and achieve the synergistic regulation of the internal and external microenvironment of the neurovascular unit (NVU).

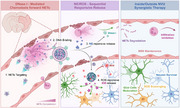

## 1 Introduction

Acute ischemic stroke (AIS) is one of the most common cerebrovascular diseases, with high mortality and severe disability.^[^ ^1^ ^]^ Despite the development of several thrombolytic treatment standards represented by recombinant tissue plasminogen activator (rt‐PA), severe secondary ischemia‐reperfusion injury leads to poor prognosis in clinical practice.^[^ ^2^ ^]^

As a key component of the cerebrovascular system, NVU suffers from the massive ROS generated by reperfusion, leading to structure and function injury. NVU homeostasis is disrupted, including neuronal apoptosis, abnormal activation of glial cells, injury of endothelial cells, loose of tight junctions, and destruction of BBB integrity.^[^ ^3^ ^]^ Although multiple antioxidant drugs, including small molecules and antioxidant enzymes, are used to alleviate reperfusion injury. Natural antioxidants such as curcumin and resveratrol, and classic model drugs such as rapamycin and fingolimod have shown various therapeutic effects, including inhibiting oxidative stress, rescuing neuronal apoptosis, and inhibiting microglial activation to initiate immune responses.^[^ ^4^ ^]^ Unfortunately, these drugs are prone to oxidation and become ineffective before reaching the lesion site. On the other hand, due to the presence of the blood‐brain barrier, although the integrity of the BBB is partially disrupted during ischemia‐reperfusion, therapeutic agents are still difficult to accumulate at an effective dose in the brain.^[^ ^5^ ^]^ Therefore, it is necessary to develop suitable drug delivery systems to protect the activity of drugs and overcome the shortcomings of openness. Polymers and metal nanoparticles are widely used to encapsulate antioxidant and anti‐inflammatory small molecules, and significantly improve delivery efficiency and therapeutic efficacy through their targeted and specific release at the lesion site.^[^ ^6^ ^]^ The use of deposited fibrin and highly expressed molecules in stressed endothelial cells for peptide and antibody modification on the surface of nanoparticles is a common active targeting method. However, the complex modifications increase the production cost and conversion difficulty of nanomedicine.

Compared to small molecule drugs, enzymes exhibit higher specificity and biological activity, providing enormous potential for disease treatment. However, their larger molecular weight and complex structure also lead to lower stability and barrier‐crossing efficiency, posing further challenges for delivery. In this context, membranes and extracellular vesicles derived from biological sources exhibit excellent protective effects. Meanwhile, nanoenzymes with various antioxidant enzyme activities, including cerium oxide (CeO_2_), Manganese dioxide (MnO_2_), and Prussian blue nanoparticles provided alternatives to catalase. The new generation of delivery materials has also evolved into functional types, such as polymer materials with ROS response and clearance functions, and dopamine coatings with antioxidant properties. The combination of this delivery function and therapeutic function not only makes the delivery system more intelligent and effective but also more elegant, which is beneficial for clinical translation.

Recently, increasing evidence reveal neutrophils’ pivotal role in ischemia‐reperfusion injury. Neutrophils could be rapidly recruited to the lesion area and overactivated to form NETs.^[^ ^7^ ^]^ The released proteases and chromatin may exacerbate neurotoxicity and inflammatory cascade reactions. Myeloperoxidase (MPO) and Neutrophil elastase (NE) are two typical NETs proteases, which degrade extracellular matrix and tight junctions, damage endothelial cells, and trigger inflammatory cascades.^[^ ^8^ ^]^ The network DNA acts as a microthrombus skeleton, capturing other blood cells and forming immunothrombosis.^[^ ^9^ ^]^ NETs play an indispensable role in mediating thrombolytic resistance, exacerbating ischemia‐reperfusion injury, and inducing hemorrhagic transformation, and recurrence.^[^ ^10^ ^]^ Thanks to its natural DNA degradation function, DNase I is used to degrade NETs. But it also faces delivery problems such as fast blood circulation clearance, short half‐life, and vulnerable activity. Several delivery systems have been developed to address these challenges. DNase I was encapsulated in sialic acid‐modified nanoparticles and effectively hitchhiked on neutrophils across the blood‐brain barrier into the injured brain parenchyma after intravenous injection.^[^ ^11^ ^]^ Coincidentally, M2‐type macrophage extracellular derived vesicles were used to encapsulate DNase I and endow it with the ability to cross the BBB.^[^ ^12^ ^]^ But these delivery strategies follow the design of conventional brain‐targeted delivery systems for stroke treatment while ignoring the unique physiological location of NETs that distinguishes them from other molecular events.

Notably, an ischemic stroke is not only a disease of the brain but also a disease of the vasculature that leads to damage of the brain.^[^ ^13^ ^]^ After reperfusion, damaged cerebral capillaries recruit white blood cells and platelets, leading to sustained inflammatory response and immune thrombosis. This sustained disturbance exacerbates the steady‐state disruption of the NVU microenvironment, ultimately leading to irreversible neuronal damage. Due to the severe pathological damage and the crosstalk of microenvironmental homeostasis in AIS, simple neuroprotective agents are not effective in clinical application. Collaborative therapy has been proven to be superior to monotherapy, including targeting multiple neuronal death mechanisms,^[^ ^14^ ^]^ inflammation,^[^ ^4^ ^]^ and metabolic microenvironment regulation.^[^ ^15^ ^]^ However these treatment strategies are still limited to events inside the NVU, namely neurons and glial cells, and pay less attention to challenges from blood vessels. Focusing on the vascular side of NVU at the lesion site, the rapidly circulating blood flow environment, and fewer identifiable abnormal molecules present new challenges for the design of delivery systems.

The biomimetic delivery strategy based on natural chemotaxis has significant advantages in delivery efficiency, accuracy, and response speed. Inspired by endogenous mechanisms, macrophages, and neutrophils are powerful tools for accurately delivering to ischemic lesions through inflammatory chemotaxis.^[^ ^11^, ^16^ ^]^ However, it is necessary to be vigilant about the inevitable inflammatory initiation and exacerbation of damage that this process may bring, as well as the potential risk of further opening up the BBB. Correspondingly, the natural low immunogenicity and high half‐life of cell membrane coatings have received widespread attention.^[^ ^17^ ^]^ However, due to the lack of support from cellular structure and signaling pathway transduction, the retention of cell membrane chemotaxis function is limited. On the other hand, this low immunogenicity refers to the self. In fact, the use of biomaterials, especially those derived from immune cells, introduces significant uncertainty and production issues for delivery systems. These issues of cellular chemotaxis have forced the focus of delivery system research to shift toward molecular chemotaxis. By leveraging the specific affinity of L‐arginine (l‐Arg) for inducible nitric oxide synthase (iNOS)/ROS, nanomotors can achieve positive chemotaxis of iNOS/ROS concentration gradients to reach the inflammatory microenvironment.^[^ ^18^ ^]^ Through asymmetric modification of enzymes, nanomotors exhibited deep penetration in the substrate concentration gradient of disease‐related regions.^[^ ^19^ ^]^ In sequential delivery, the local release of enzymes was also used to clear delivery barriers.^[^ ^20^ ^]^ These explorations expanded the application of enzymes in drug delivery systems but were still limited to their catalytic effects. The driving force of enzymes has been reported,^[^ ^21^ ^]^ but there is no suitable application in the disease microenvironment.^[^ ^22^ ^]^ We propose that enzymes themselves may have the potential to become excellent targeting functional groups, utilizing their chemotaxis and substrate interactions to achieve enrichment at specific sites. The rapid response mechanism and unique intravascular localization of NETs make them potential guiding molecules for the AIS lesions as well as the next generation of AIS nanomedicines.

**Scheme 1:**
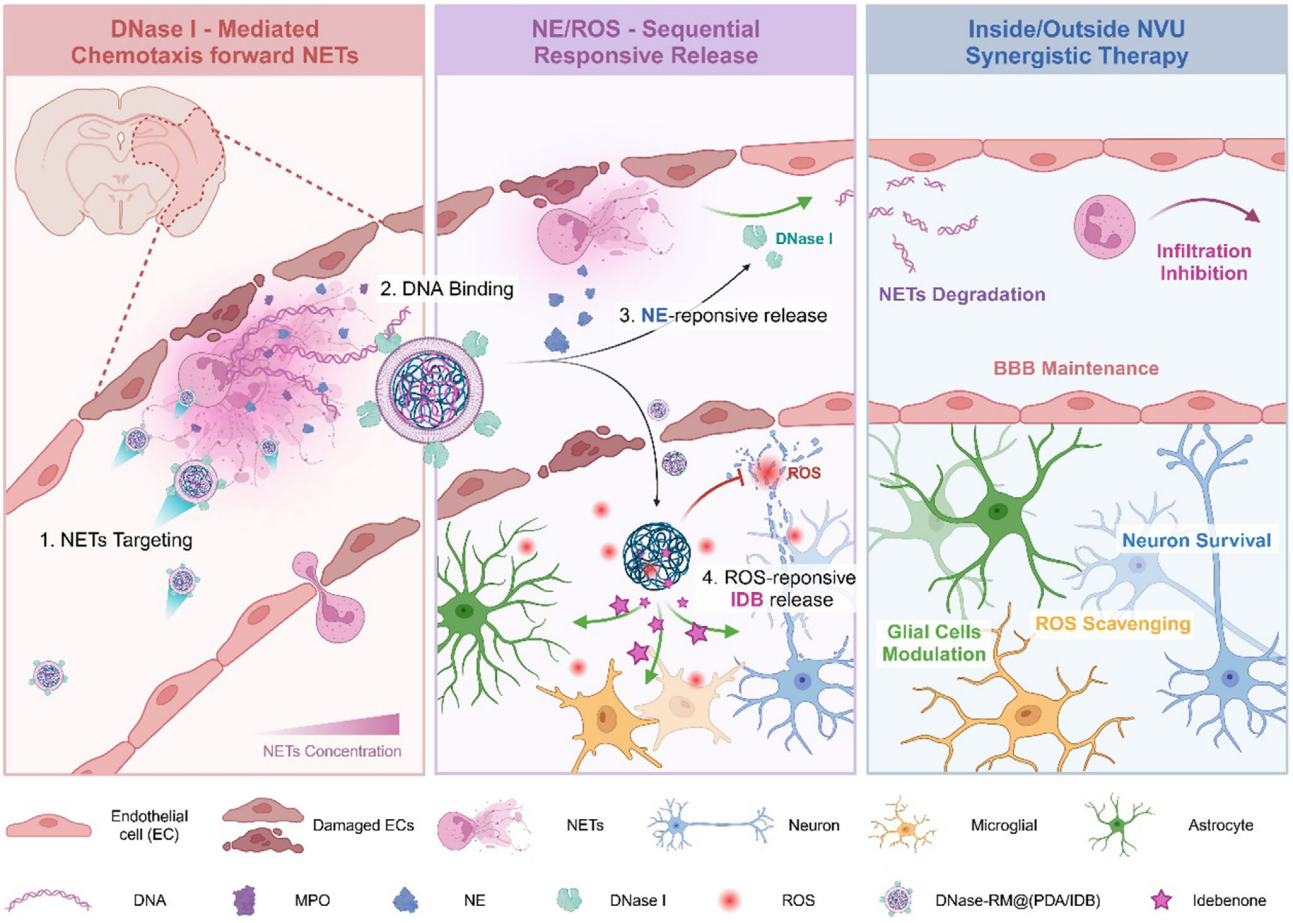
Schematic illustration of targeted, responsive, and therapeutic mechanism of DNase‐RM@(PDA/IDB) in cerebral ischemia‐reperfusion injury. Nanoparticles accumulate in the blood vessels of NETs‐rich lesion areas through DNase I‐mediated chemotaxis. Subsequently, they are cleaved by NE in NETs to release DNase I, which degrades harmful NETs, protects vascular endothelial cells, and reduces subsequent neutrophil infiltration. Meanwhile, the remaining nanoparticles penetrate the brain parenchyma through the compromised BBB, consuming excessive ROS and releasing IDB. The dual antioxidant and anti‐inflammatory effects by PDA and IDB effectively rescue neuronal apoptosis and reduce glial cell activation. The therapeutic strategy of synergistically regulating the intravascular and brain parenchyma of ischemia‐reperfusion sites comprehensively regulates the both inside and outside NVU microenvironment. Created in https://BioRender.com.

Herein, we propose that DNase I may have the potential to become potent targeting functional groups, as a concept of proof. Specifically, conjugated DNase I was used to target nanoparticles to ischemia‐reperfusion areas rich in NETs (**Scheme** **1**). DNase I was connected to the surface of dopamine nanoparticles coated on red blood cell membranes through a cleavable peptide, achieving extended circulation time. On the strength of the concentration gradient chemotaxis toward DNA and interaction with DNA, nanoparticles were enriched in the lesion area. The cleavage of highly expressed neutrophil elastase (NE) released DNase I to degrade pathologic NETs, as well as allowed nanoparticles to enter the brain through damaged BBB for multi‐target therapy. The integration of therapeutic and targeted functions of enzymes simplified the design and preparation of drug delivery systems, improving their efficacy and clinical translational value. Considering the important role of NETs in the development process of various diseases like inflammation and tumor,^[^ ^23^ ^]^ this concept we first proposed is expected to become a universal nanodrug targeting strategy in the future.

## 2 Results

### 2.1 Preparation and Characterizations of DNase‐RM@(PDA/IDB)

**Figure 1:**
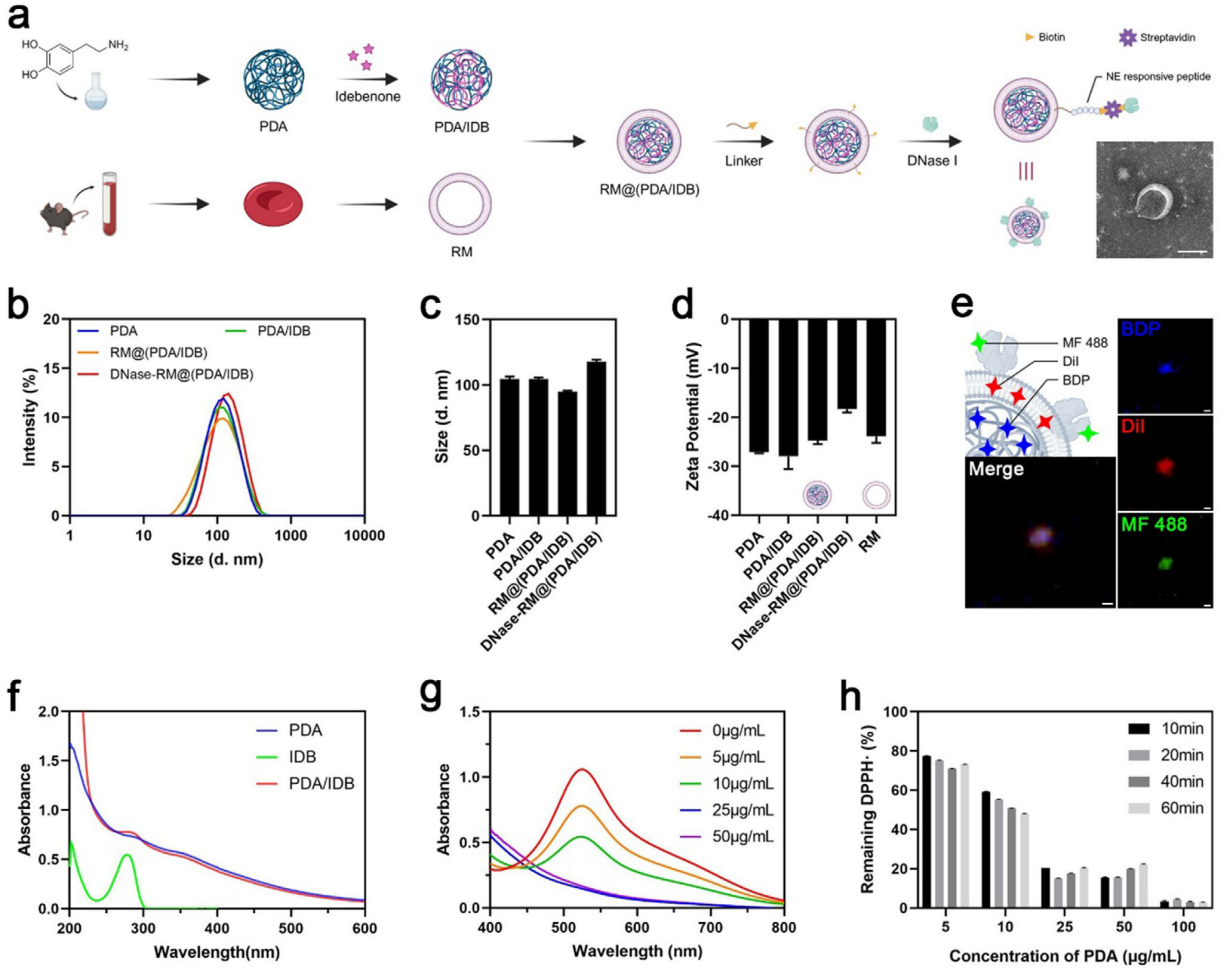
Characterization of DNase‐RM@(PDA/IDB). a) Schematic depicting the preparation and composition of DNase‐RM@(PDA/IDB). DNase I is modified on the surface of main nanoparticle through an NE‐responsive peptide. Scale bar, 100 nm. b,c) Size distribution of PDA, PDA/IDB, RM@(PDA/IDB), and DNase‐RM@(PDA/IDB). d) Zeta potential of PDA, PDA/IDB, RM@(PDA/IDB), DNase‐RM@(PDA/IDB), and RM. e) Representative super‐resolution images of fluorescence probes labeled DNase‐RM@(PDA/BDP). Scale bars, 200 nm. f) UV‐vis absorbance spectra of PDA, IDB, and PDA/IDB. g,h) UV‐vis absorbance spectra and remaining percentage of DPPH• after incubation with different concentrations of PDA. Data are presented as mean ± SD (n = 3 independent experiments). Illustrations were created in https://BioRender.com.

The preparation of DNase‐RM@(PDA/IDB) is shown in **Figure** **1**a. First, biocompatible and biodegradable polydopamine (PDA) nanoparticles were obtained through oxidative polymerization. Idebenone (IDB) is loaded through π‐π stacking interaction with PDA and subsequently purified by centrifugation and ultrafiltration to remove the free fraction. UV‐vis scanning showed successful drug loading (Figure 1f), and HPLC determined the drug loading (DL) to be 12.9%. The mouse red blood cell membrane (RM) was extracted and encapsulated on the surface of nanoparticles to endow it with in vivo long‐circulating properties. The modification of DSPE‐PEG2000‐AAPVK‐biotin provided an NE cleavable binding site on the surface of the nanoparticles (Figure S1, Supporting Information). The biotin‐avidin system is a mild and efficient reaction, effectively protecting the bioactivity of materials. Dynamic light scattering (DLS) showed that the hydrodynamic diameters of different formulations were ≈100 nm (Figure 1b,c). To determine the construction of each component, we labeled PDA nanocore, red blood cell membrane, and DNase I with three different probes. The super‐resolution confocal images displayed the co‐localization, confirming successful synthesis (Figure 1e). In addition, the ROS scavenging ability of PDA nanoparticles was investigated in vitro. 2,2‐Diphenyl‐1‐picrylhydrazyl (DPPH•) is a stable free radical often used to evaluate the free radical scavenging activity of antioxidant materials. After incubation with PDA nanoparticles of different concentrations, a single electron of DPPH• was captured, leading to the decrease in absorbance level, with concentration dependence and time dependence (Figure 1g,h). Therefore, we speculated that PDA nanoparticles could scavenge ROS and preserve the stability of idebenone in circulation.

### 2.2 DNase I‐Mediated Chemotaxis Forward NETs

**Figure 2:**
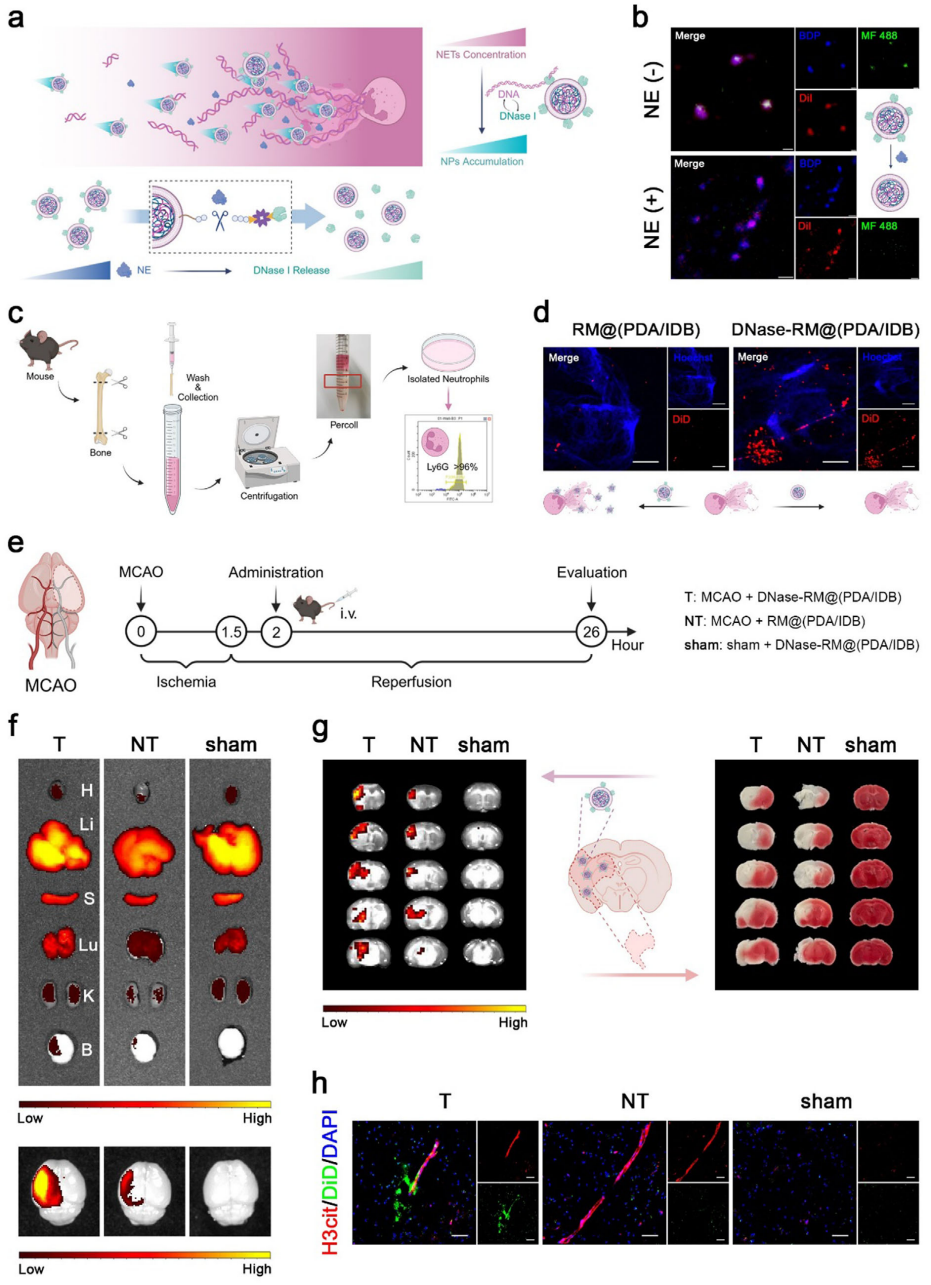
NETs binding and targeting ability of DNase‐RM@(PDA/IDB) in vivo. a) Schematic diagram of DNase I‐mediated accumulation and NE‐induced release of DNase‐RM@(PDA/IDB). b) Representative super‐resolution images of fluorescence probes labeled DNase‐RM@(PDA/BDP) incubated with NE (1 µg mL^−1^) for 24 h. Scale bars, 500 nm. c) Schematic depicting the extraction and isolation of primary neutrophils. d) Representative confocal microscopic images of the adhesion of DiD‐labeled RM@(PDA/BDP) and DNase‐RM@(PDA/BDP) on NETs after 15 min incubation. Scale bars, 10 µm. e) Illustration and timeline of MCAO model establishment, drug treatment, and therapeutic evaluation. f) Representative ex vivo IVIS images of major organs 24 h after intravenous injection. g) Representative IVIS fluorescence images and TTC staining images of brain slices. h) Distribution of DiD signals and NETs. Scale bars, 100 µm. Illustrations were created in https://BioRender.com.

After ischemic stroke, neutrophils quickly recruit to the lesion site and release NETs, which may serve as an intravascular guiding molecule. The main components of NETs are DNA scaffolds and various harmful proteins, which need to be cleared as soon as possible to inhibit secondary injury and inflammatory recruitment reactions. The interaction between DNase‐RM@(PDA/IDB) and NETs is mainly mediated by two molecular events (**Figure** **2**a). First, the chemotactic effect of DNase I on the substrate DNA provided targeting. To validate this concept, we extracted primary neutrophils from mouse bone marrow and induced NETs formation in vitro (Figure 2c). Compared to unmodified nanoparticles, DNase I‐modified nanoparticles bound more to in vitro NETs after short incubation (Figure 2d). This suggested that in addition to playing a catalytic role, DNase I can also serve as a targeted ligand to guide the accumulation of nanoparticles at the lesion site. After reaching the rich NETs region, the sensitive sequence AAPV would be cleaved by highly expressed NE (Figure S3, Supporting Information), releasing DNase I and the main nanoparticles to exert their functions. To verify enzyme cleavage sensitivity, fluorescently labeled nanoparticles were incubated with NE. The disappearance of the green signal indicated that DNase I has dissociated from the nanoparticles and cannot be detected by super‐resolution confocal microscopy (Figure 2b).

Subsequently, a transient middle cerebral artery occlusion/reperfusion (MCAO) mice model was established to further investigate the in vivo targeting of DNase‐RM@(PDA/IDB) (Figure 2e). Fluorescently labeled DNase‐RM@(PDA/IDB) and RM@(PDA/IDB) were injected into sham or MCAO mice respectively. Since BBB disruption after ischemia‐reperfusion allows leakage of vascular contents into the brain, fluorescent signals were observed in the brains of MCAO mice. DNase‐RM@(PDA/IDB) showed stronger brain accumulation by interacting with NETs (Figure 2f; Figure S4, Supporting Information). Notably, triphenyltetrazolium chloride (TTC) staining demonstrated the colocalization of fluorescence signal and the area of infarction, further indicating the selective accumulation of DNase‐RM@(PDA/IDB) around the lesions (Figure 2g).

Encouraged by the increased retention at the lesions, we continued to explore whether DNase‐RM@(PDA/IDB) could efficiently enter the brain parenchyma. Immunofluorescence staining of blood vessels or NETs was performed on brain sections of mice injected with fluorescently labeled nanoparticles to investigate their distribution in the brain. Similar to the IVIS results, the strongest fluorescence signal was observed in MCAO mice injected with DNase‐RM@(PDA/IDB). Moreover, the fluorescence of the nanoparticles was widely distributed in the parenchyma surrounding microvessels labeled by CD34 (Figure S5, Supporting Information). In addition, we observed that DNase‐RM@(PDA/IDB) partially colocalized with the NETs marker Citrullinated histone H3 (H3cit) in microvessels and permeated nearby parenchyma (Figure 2h; Figure S6, Supporting Information). However, a similar association was not observed in the RM@(PDA/IDB) group. These results indicated that DNase I mediated the orientation and binding of the nanoparticles to NETs, enhanced adhesion, and retention at the lesion site, and allowed the nanoparticles to enter the brain parenchyma through the damaged BBB for further neuroprotection and microenvironmental regulation.

### 2.3 NETs Degradation and Inhibition In Vitro

**Figure 3:**
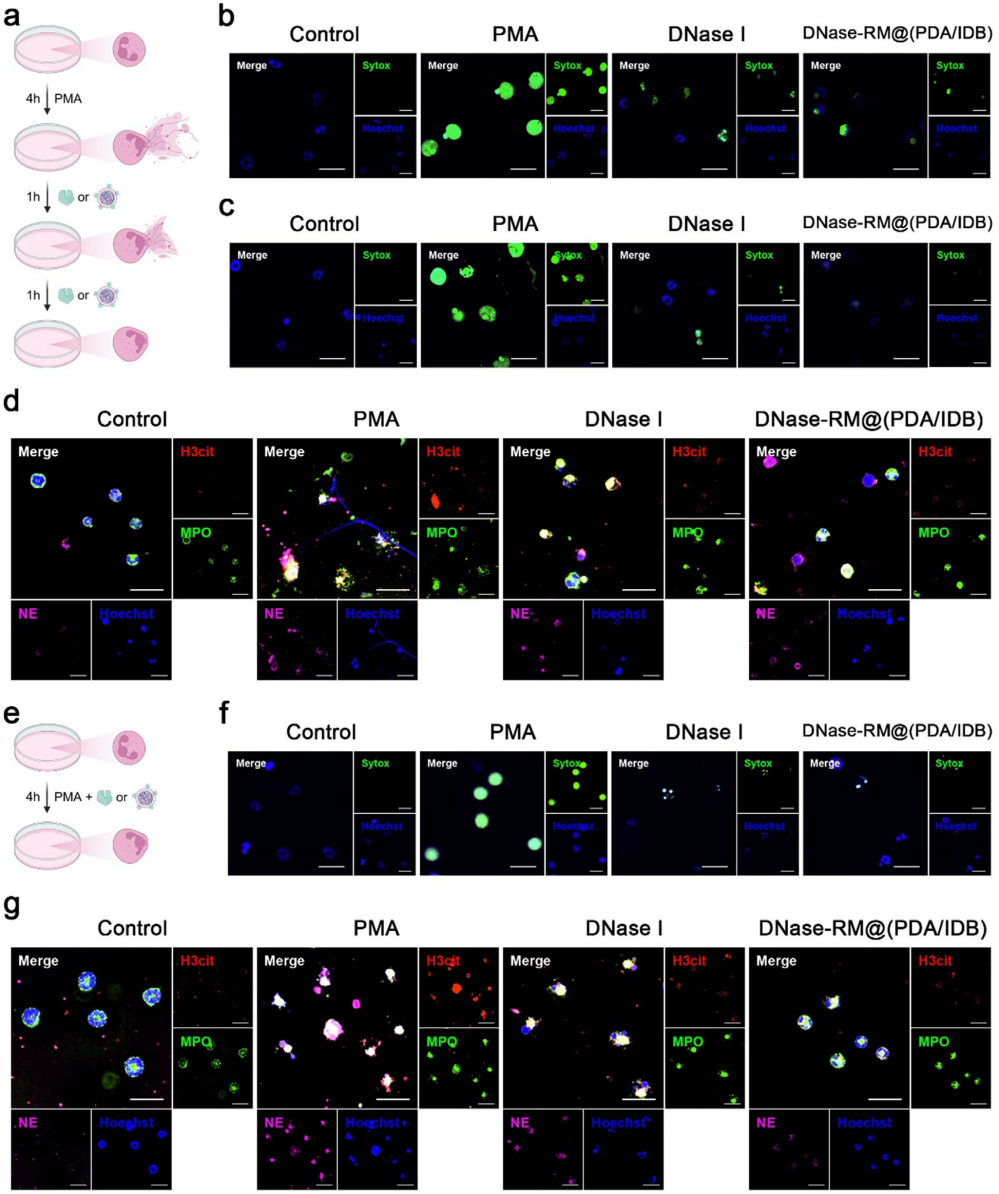
NETs degradation and inhibition in vitro. a) Schematic depicting the process of NETs formation and degradation. b,c) Representative confocal microscopic images of DNA in live or dead cells after b) 1 h or c) 2 h incubation. Scale bars, 20 µm. d) Representative confocal microscopic images of NETs markers after 2 h incubation. Scale bars, 20 µm. e) Schematic diagram of the process of NETs inhibition. f) Representative confocal microscopic images of DNA in live or dead cells. Scale bars, 20 µm. g) Representative confocal microscopic images of NETs marker. Scale bars, 20 µm. Illustrations were created in https://BioRender.com.

To investigate the efficient clearance of NETs by DNase‐RM@(PDA/IDB), an in vitro NETs degradation assay was performed with primary neutrophils (**Figure** **3**a). Sytox Green, a DNA probe that does not penetrate the membrane of living cells, was used to indicate released NETs scaffolds. After stimulation of neutrophils with Phorbol 12‐myristate 13‐acetate (PMA), extensive extracellular DNA signals were observed. Free DNase I or DNase‐RM@(PDA/IDB) degraded NETs and significantly reduced extracellular DNA structures within 1 h (Figure 3b). After incubation for 2 h, the green fluorescence signal basically disappeared (Figure 3c). Cellular immunofluorescence staining was used to further investigate NETs markers. H3cit, MPO, and NE are released and decorated on DNA during the NETs formation, which causes continuous damage to endothelial cells and induces the activation and aggregation of platelets and immune cells. PMA stimulation significantly increased the expression of the three markers, with scattered distribution around DNA. After incubation with DNase I or DNase‐RM@(PDA/IDB), the free signals in the background faded and overall fluorescence levels of H3cit and NE decreased (Figure 3d). These results indicated that DNase‐RM@(PDA/IDB) retained the ability of DNase I to remove NETs efficiently. Next, PMA stimulation and DNase I or DNase‐RM@(PDA/IDB) were given simultaneously to investigate the inhibitory effect on the NETs formation (Figure 3e). Compared with the untreated negative control group, both extracellular DNA and NETs markers were significantly reduced, demonstrating the timely inhibition of NETs (Figure 3f,g).

### 2.4 Neuroprotection Based on ROS Scavenging and Mitochondrial Homeostasis In Vitro

**Figure 4:**
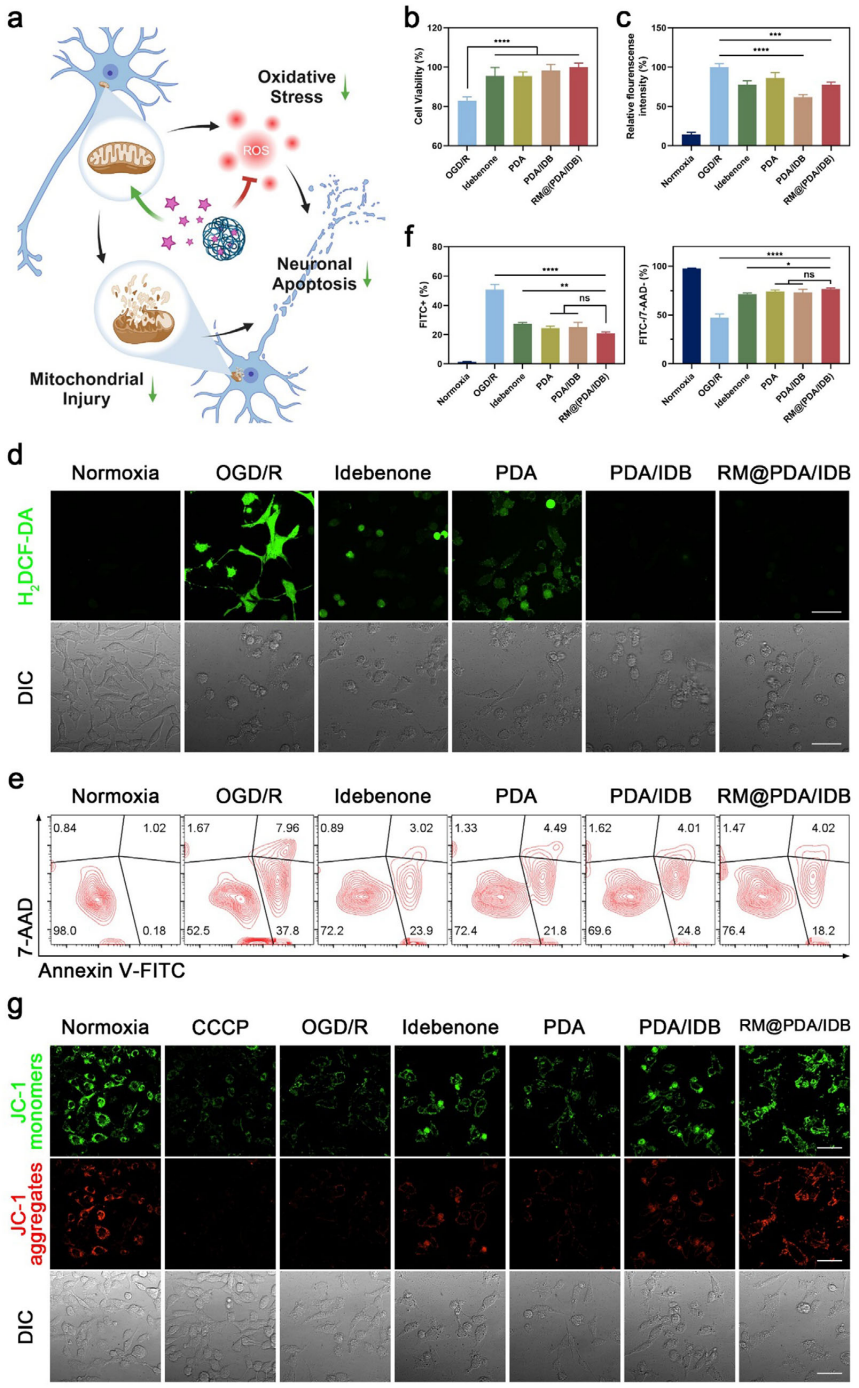
Neuroprotection of RM@(PDA/IDB) via ROS scavenging and mitochondrial membrane potential maintaining in OGD/R models in vitro. a) Schematic diagram of the synergistic anti‐apoptosis effect in neurons. b) The cell viability of OGD/R treated cells after incubation with different formulations for 24 h. c) Flow cytometry analysis of ROS probe DHE fluorescence intensity in OGD/R treated cells. d) Representative confocal microscopic images of ROS probe H2DCF‐DA in OGD/R treated cells. Scale bars, 50 µm. e,f) Flow cytometry analysis of OGD/R induced cell apoptosis gating on Annexin V‐FITC/7‐AAD staining. g) Representative confocal microscopic images of mitochondrial membrane potential probe in OGD/R treated cells. Scale bars, 50 µm. All Data are presented as mean ± SD. (n = 3 independent experiments). Statistical analysis was performed by one‐way ANOVA with Tukey post‐test. ns not significant, *p < 0.05, **p < 0.01, ***p < 0.001 and ****p < 0.0001. Illustrations were created in https://BioRender.com.

During ischeme‐reperfusion (I/R), excessive ROS generation causes direct oxidative damage to cellular structures such as DNA, proteins, and lipids. On the other hand, as an important site of cellular energy metabolism, mitochondria suffer oxidative stress and dysfunction. The combination of structural and functional dysregulation leads to neuronal apoptosis, often described as irreversible damage by reperfusion (**Figure** **4**a). Therefore, we established an oxygen glucose deprivation/re‐oxygenation (OGD/R) model in SH‐SY5Y cells to explore the neuroprotective mechanism of RM@(PDA/IDB). After OGD/R treatment, neuronal cell viability was reduced and could be rescued by free idebenone, PDA, PDA/IDB, and RM@(PDA/IDB) treatment (Figure 4b). To investigate the level of oxidative stress, two fluorescent probes were used to detect intracellular ROS. Compared with the OGD/R group, both free idebenone and PDA reduced intracellular ROS and showed a synergistic effect (Figure 4c,d). This neuroprotective effect was supported by the inhibition of apoptosis by flow cytometry (Figure 4e,f). Mitochondrial membrane potential (MMP) is necessary for the maintenance of normal physiological activities of mitochondria, closely related to the energy supply and survival state. JC‐1 is an MMP indicator. Under normal conditions, JC‐1 accumulates in mitochondria in a potentially dependent manner and emits red fluorescence. After OGD/R, the mitochondria depolarized and the red fluorescence signal disappeared, as similar results obtained after treatment with the mitochondrial electron transport chain inhibitor Carbonyl cyanide 3‐chlorophenylhydrazone (CCCP). The MMP in the treatment group containing idebenone was restored, indicating the mitochondrial protection mediated by idebenone (Figure 4g). Taken together, RM@(PDA/IDB) protected neurons against I/R injury by scavenging ROS to alleviate oxidative stress and maintain mitochondrial function.

### 2.5 Brain Damage Rescue in MCAO Mouse Model

**Figure 5:**
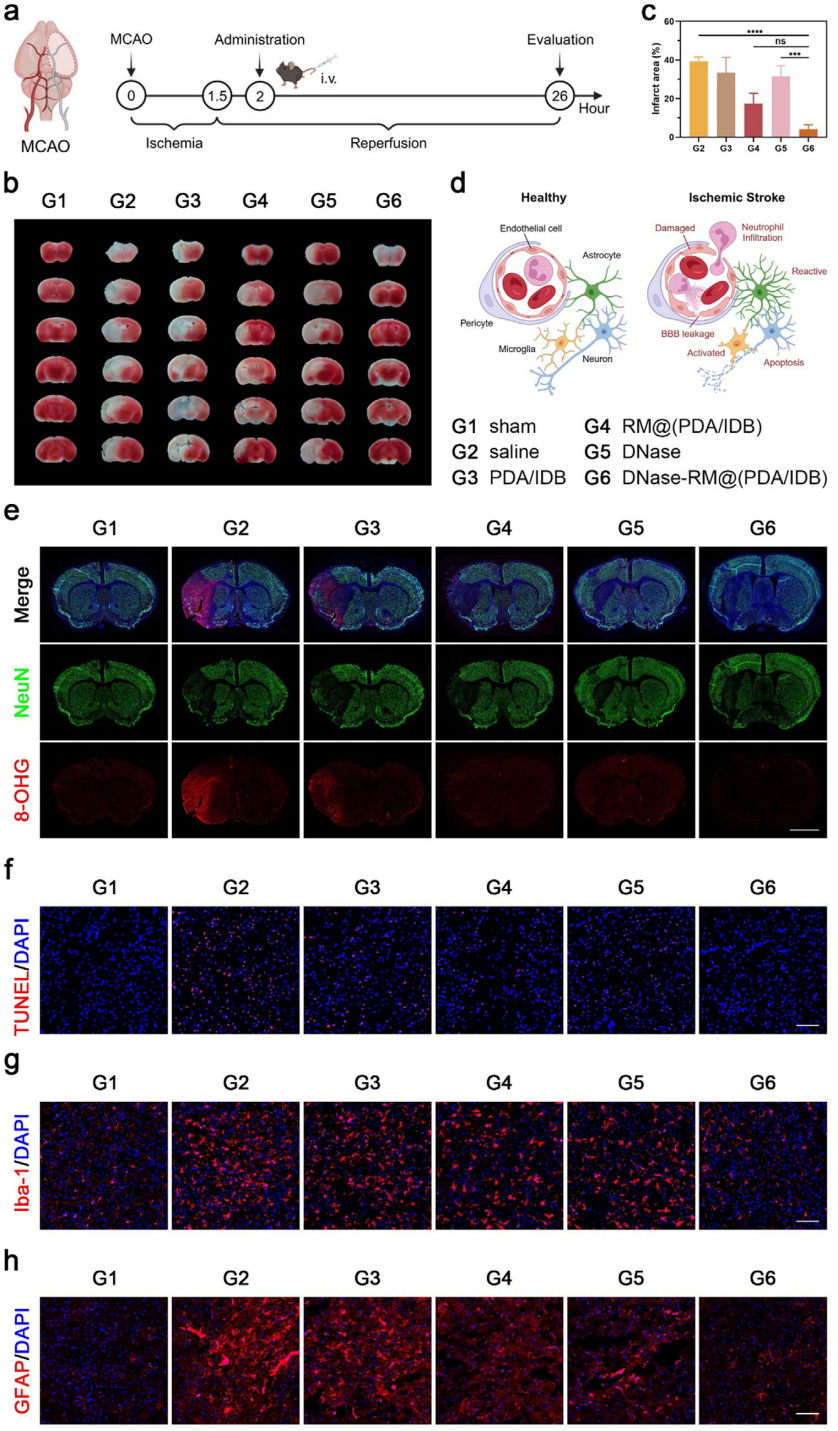
Therapeutic effect of DNase‐RM@(PDA/IDB) in MCAO mice model. a) Illustration and timeline of MCAO model establishment, drug treatment, and therapeutic evaluation. b) Representative TTC staining images of brain slices. c) Semi‐quantified results of TTC staining in b). Data are presented as mean ± SD (n = 3 independent experiments). Statistical analysis was performed by one‐way ANOVA with Tukey post‐test. ns not significant, ***p < 0.001 and ****p < 0.0001. d) Schematic diagram of NVU in healthy and ischemic stroke. e) Representative fluorescence images of nerve injury and oxidative stress in MCAO model mice treated with different formulations. Scale bars, 2 mm. f) Representative fluorescence images of TUNEL staining in the ischemic penumbra. Scale bar, 100 µm. g,h) Representative fluorescence images of g) microglia and h) astrocyte activation. Scale bars, 100 µm. Illustrations were created in https://BioRender.com.

Based on the above results, we then evaluated the therapeutic effect of DNase‐RM@(PDA/IDB) on ischemia‐reperfusion injury in MCAO mouse model (**Figure** **5**a). The complete formulation group integrated DNase I and the main nanoparticle showed the greatest protection. Specifically, the infarct size indicated by TTC staining decreased from ≈40% to ≈5% (Figure 5b,c; Figure S8, Supporting Information). Moreover, oxidative stress injury indicated by immunostaining of 8‐hydroxyguanosine (8‐OHG) reduced after DNase‐RM@(PDA/IDB) treatment while the expression of neuron marker (NeuN) increased synchronously (Figure 5e; Figure S9, Supporting Information). In addition, TUNEL‐labeled apoptosis was also alleviated (Figure 5f; Figure S10, Supporting Information). These results supported that DNase‐RM@(PDA/IDB) could effectively rescue the ischemic penumbra, reduce neuronal damage, and preserve basic brain function.

### 2.6 NVU Microenvironment Regulation

NVU is the fundamental unit of brain structure and function and has been widely considered for its important function in healthy and ischemic stroke (Figure 5d). Here, we examined multiple mechanisms by which DNase‐RM@(PDA/IDB) regulates the NVU microenvironment. Microglia and astrocytes are the main immune cells residing in the brain. Abnormal activation of glial cells after ischemia‐reperfusion aggravates nerve injury through inflammatory response. Immunofluorescence staining showed significant activation of microglia and astrocytes in the ischemic penumbra of MCAO model mice (Figure 5g,h; Figures S11, S12, Supporting Information). Specifically, microglia changed from a branching phenotype to an amoebic morphological appearance, while expression of glial fibrillary acidic protein (GFAP), an astrocyte activation marker, was upregulated. In contrast, DNase‐RM@(PDA/IDB) could attenuate activation to normal levels.

### 2.7 NETs Degradation and BBB Remodeling in MCAO Mouse Model

**Figure 6:**
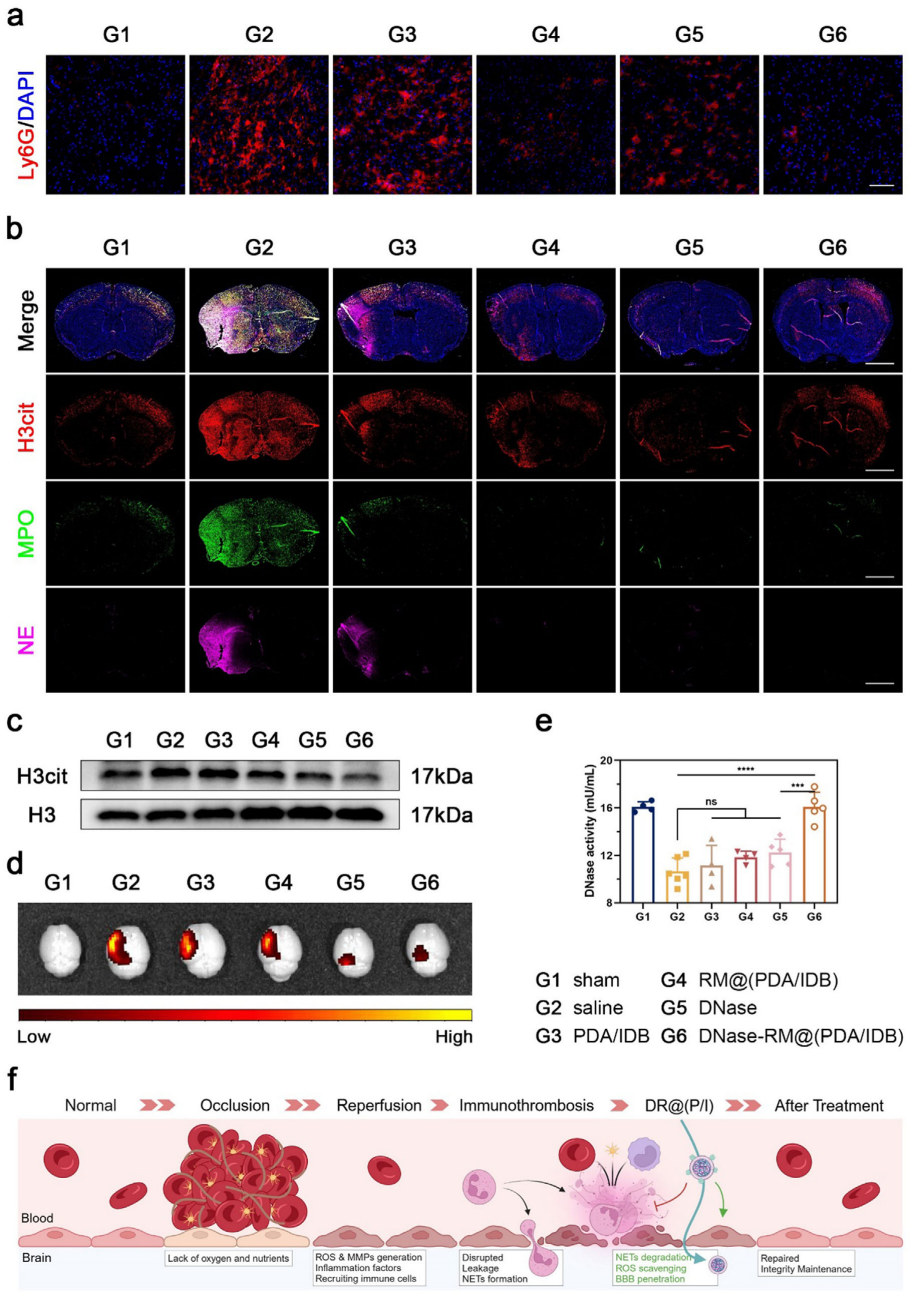
NETs degradation and BBB protection in vivo. a) Representative fluorescence images of neutrophil infiltration in the ischemic penumbra of MCAO model mice treated with different formulations. Scale bar, 100 µm. b) Representative fluorescence images of NETs markers. Scale bars, 2 mm. c) Western blotting of H3cit expression. d) Representative ex vivo IVIS images of Evans Blue leakage. e) DNase activity from MCAO model mice blood. Data are presented as mean ± SD. Statistical analysis was performed by one‐way ANOVA with Tukey post‐test. ns not significant, ***p < 0.001 and ****p < 0.0001. f) Schematic depicting the process of BBB injury during ischemic‐reperfusion and treatment. Illustrations were created in https://BioRender.com.

In addition to resident glial cells, the brain is also infiltrated by peripheral immune cells after ischemic stroke. Neutrophils are the first to arrive and destroy BBB integrity by forming NETs, initiating an inflammatory cascade, and exacerbating damage to the lesion site (Figure 5d). Therefore, we hypothesized that DNase‐RM@(PDA/IDB) could reduce neutrophil recruitment and maintain BBB homeostasis. First, neutrophil infiltration indicated by immunostaining of Ly6G reduced in the ischemic penumbra (**Figure** **6**a; Figure S13, Supporting Information). Subsequently, NETs formation in the brain was observed using immunofluorescence staining of NETs markers (Figure 6b; Figure S14, Supporting Information). A significant NETs distribution was observed in the ischemic hemisphere of MCAO model mice compared with the sham group. After treatment, the distribution of NETs decreased and the overall brightness reduced. Notably, NETs were almost no longer observed after DNase‐RM@(PDA/IDB) degradation. Westing blot results similarly supported the efficient NETs clearance by DNase‐RM@(PDA/IDB) (Figure 6c). Correspondingly, treatment with DNase‐RM@(PDA/IDB) restored mouse plasma DNase I activity (Figure 6e) and reduced brain Evans Blue leakage (Figure 6d). The above results supported the excellent efficacy of DNase‐RM@(PDA/IDB) in NETs degradation and BBB integrity maintenance (Figure 6f).

### 2.8 Biosafety In Vitro and In Vivo

Finally, we evaluated the biocompatibility of DNase‐RM@(PDA/IDB). All components of DNase‐RM@(PDA/IDB) were biodegradable materials and no cytotoxicity was observed (Figure S7, Supporting Information). Hemolysis assay showed that DNase‐RM@(PDA/IDB) has good blood compatibility (Figure S15, Supporting Information). Moreover, hematoxylin‐eosin (H&E) staining of tissue sections indicated that DNase‐RM@(PDA/IDB) treatment did not cause organ toxicity (Figures S16, S17, Supporting Information). Besides, the results of liver and kidney function markers in different treated mice were all within the normal range (Figure S18, Supporting Information). Taken together, these results demonstrated that DNase‐RM@(PDA/IDB) possessed fine in vitro and in vivo biosafety, which was critical for the clinical application.

## 3 Discussion

Current nano‐strategies for the treatment of ischemic stroke mainly focus on two directions: enhancing thrombolytic efficacy and reducing reperfusion injury. However, there is a vacancy of research on the changes in the blood vessels during reperfusion and the corresponding regulation strategy. Actually, blood vessels play a crucial role in all stages of the AIS pathological process. As a metabolic interface, blood vessels support substance exchange, regulate permeability to maintain selective barriers, regulate cell transport to control immune responses, control endothelial cell function through biomechanical signal transduction, and connect systemic organs.^[^ ^13^ ^]^ Recently, NETs have emerged as promising targets due to their inherent property of rapidly participating in immune‐inflammatory regulation. Unfortunately, attention to NETs remains confined to the brain. This means using NETs formation as a brain entry mechanism or using traditional cross‐BBB strategies to deliver NETs therapeutic agents. The persistent secondary injury of intravascular NETs to endothelial cells and the recruitment of immune inflammation and immune thrombosis have rarely received appropriate treatment strategies. In this study, we focused on NETs in blood vessels after ischemic stroke to explore the influence of their unique location on the external origin of NVU. Accordingly, strategies for co‐regulation inside and outside the NVU also pose new challenges for the design of nano‐delivery systems.

Continuous blood flow provides basic support for efficient material exchange and mechanical stress homeostasis. At the same time, the strong flow shear force and weak accumulation of abnormal material make the retention of nanoparticles in diseased blood vessels more challenging. Enzyme‐driven nanomotors have shown great potential for chemotaxis and penetration at lesion sites due to their powerful power. The utilization of the driving force of the enzyme mostly depends on its catalytic production of gases, such as O_2_, NO, and CO_2_. Enzyme chemotaxis has been studied for a long time, but it has not been fully and properly applied due to the limitation of non‐biocompatible/non‐physiological concentration/non‐disease related fuels.^[18,21c,24]^ In this study, we exploited the chemotaxis of DNase I to the DNA backbone in NETs to navigate nanoparticles to the lesion site and achieve the integration of catalytic therapy and targeting functions. To our knowledge, this is the first demonstration of enzyme‐mediated targeting in vivo. Given the findings of NETs in a variety of diseases, it may become a universal chemotactic targeted delivery strategy.

Compared with existing NETs‐targeting strategies, enzyme chemotaxis‐mediated targeting has the following advantages: (1) No inflammatory chemotaxis processes involved, avoiding the exacerbation of NETs production and secondary damage caused by the most common neutrophil hitchhiking strategy; (2) Taking into account the NETs in blood vessels, addressing the limitations of traditional cross BBB strategies in lesion localization, and expanding the scope of treatment; (3) Integrating enzyme targeting and therapeutic effects, simplifying the design and preparation of delivery systems.

Finally, the high risk of recurrent vascular events after ischemic stroke is also an important factor in its poor prognosis. Recent studies suggest that increased circulating cell‐free DNA from neutrophils may be a potential mechanism for vascular risk and recurrent stroke.^[^ ^25^ ^]^ Therefore, the removal of intravascular NETs and restoration of plasma DNase activity in the present study provide promising efforts for unprevented events.

## 4 Conclusion

In this work, we report a NETs‐targeting nanoparticle based on enzyme chemotaxis, which ameliorated reperfusion injury in ischemic stroke through synergistic regulation of the internal and external microenvironment of the NVU. Under the modification of DNase I, the nanoparticles targeted NETs generated at the lesion site and performed NETs degradation and oxidative stress and inflammation regulation functions at different locations. After reaching the ischemic penumbra through the disrupted BBB, RM@(PDA/IDB) reduced neuronal apoptosis by alleviating oxidative stress and maintaining mitochondrial function. Moreover, DNase‐RM@(PDA/IDB) played a multifunctional therapeutic role in degrading NETs, maintaining BBB integrity, inhibiting neutrophil infiltration and scavenging ROS, rescuing neurons and inhibiting glial activation, and realizing the overall remodeling of the lesion microenvironment in the MCAO mouse model. This work reexamines the implications of the unique geographical location of the intravascular distribution of NETs for the design of delivery systems during the ischemia‐reperfusion phase and suggests that synergistic regulation within and outside the NVU provides new insights as an extension of overall therapeutic targets. What's more, the concept of enzyme‐mediated targeting was tested for the first time and could become a universal chemotactic targeted delivery strategy.

## 5 Experimental Section

### Materials

Dopamine hydrochloride was purchased from Energy Chemical (Shanghai, China). Maleimide‐1,2‐distearoyl‐sn‐glycero‐3‐phosphoethanolamine‐N‐[methoxy(polyethylene glycol)‐2000] (DSPE‐PEG2000‐MAL) was purchased from Xi'an ruixi Biological Technology Co., Ltd (Xi'an, China). Sulfhydryl and biotin‐modified peptide (sequence: HS‐AAPVK‐biotin) was synthesized by ChinaPeptides (QYAOBIO) (Shanghai, China). Idebenone, biotin succinimide ester (NHS‐biotin), bovine serum albumin (BSA), cell membrane probe DiI and DiD, enhanced cell counting kit‐8 (CCK‐8), and Annexin V‐FITC/7‐AAD apoptosis detection kit were purchased from Dalian Meilun Biotechnology Co., Ltd (Dalian, China). Enhanced mitochondrial membrane potential assay kit with JC‐1, Bradford protein assay kit, QuickBlock blocking buffer and primary antibody dilution buffer for western blot and immunol staining, cell lysis buffer for Western and IP, RIPA lysis buffer were purchased from Beyotime (Shanghai, China). Streptavidin was purchased from Yeasen (Shanghai, China). 2,2‐biphenyl‐1‐picrylhydrazine (DPPH), deoxyribonuclease I (DNase I), Triphenyltetrazolium chloride (TTC), and phobosol 12‐tetradecanoate 13‐acetate (PMA) were purchased from Sigma‐Aldrich (St. Louis, USA). Other chemicals were purchased from Sinopharm Chemical Reagent Co., Ltd (Shanghai, China). H2DCFDA (ROS probe), was purchased from Thermo Fisher Scientific Co., Ltd. (Shanghai, China). DNase I assay kit was purchased from Abcam (Shanghai, China). The monofilament nylon threads with silica gel coated distal end (diameter of the distal end: 0.12±0.01 mm, length: 25–30 mm) were purchased from Cinotech (Beijing China). Matrigel was purchased from BD Biosciences (San Jose, USA).

### Synthesis of PDA

The PDA nanoparticles were synthesized by the oxidative polymerization of dopamine. Briefly, 9 mL deionized water, 4 mL ethanol, and 0.8 mL ammonium hydroxide were stirred together at 25 °C for 30 min. Then 50 mg dopamine hydrochloride was added and the stirring was continued for another 24 h. The PDA was purified by the centrifugal filter unit (Mw = 100 kDa) to remove unconverted monomer. After that, the PDA solution was filtered by 0.22 µm filter membrane to obtain the PDA nanoparticles. The concentration of PDA was determined by lyophilization.

### Red blood Cell Membrane

Primary red blood cells were isolated from mouse blood. Low osmolarity solution was used to disrupt red blood cells, and the membrane was purified by repeated centrifugation.

### Cell Lines

Human neuroblastoma SH‐SY5Y cells were grown in Dulbecco's modified Eagle medium/F12 (DMEM/F12) containing 10% fetal bovine serum (FBS), 100 mg mL^−1^ streptomycin and 100 U mL^−1^ penicillin. Cells were incubated at 37 °C under a saturating humidity atmosphere containing 5% CO_2_.

### Oxygen Glucose Deprivation/Reoxygenation (OGD/R) Model

The SH‐SY5Y cells were plated in 96‐well plates (5000 cells/well), or 24‐well plates (5 × 10^4^ cells/well) and cultured at 37 °C for 24 h. The cells were then washed with DPBS, and the culture medium was then replaced with glucose‐free Hank's solution. The cells were cultured in a sealed chamber equipped with an AnaeroPack (Mitsubishi Gas Chemical, Tokyo, Japan) to maintain an anaerobic atmosphere. The cells were reoxygenated and supplemented with DMEM/F12 complete medium after OGD for 6 or 8 h in cell apoptosis assay.

### Cell Viability Assay

After OGD/R treatment, the cells were washed, and 100 µL CCK‐8 solution (10%) was added. The cells were incubated for another 2 h at 37 °C, and the 450 nm absorbance of each well was measured using a microplate reader for calculating the cell viability in each well.

### Cell Apoptosis Assay

After OGD/R treatment, cell apoptosis was detected by staining with Annexin VFITC and 7‐AAD, and analyzed by flow cytometry.

### Mitochondrial Membrane Potential Assay

JC‐1 was utilized to detect the level of mitochondrial membrane potential. After OGD/R treatment, cells were washed three times and incubated with JC‐1 probe for 30 min. Then, cells were washed and the fluorescence changes were monitored at Ex/Em = 490/525 nm and 540/595 nm with a confocal microscope.

### Primary Neutrophils

Mouse bone marrow‐derived neutrophils were isolated by density gradient centrifugation. Total bone marrow cells were collected from tibias and femurs. Primary neutrophils were obtained by centrifugation with Percoll and the RBCs were lysed. Primary neutrophils were grown in Roswell Park Memorial Institute (RPMI) 1640 medium containing 10% fetal bovine serum (FBS), 100 mg mL^−1^ streptomycin, and 100 U/mL penicillin. Cells were incubated at 37 °C under a saturating humidity atmosphere containing 5% CO_2_. Primary neutrophils were cultured in 1640 medium containing 1% BSA and 500 nm PMA for 4 h for NETs generation.

### NETs Adhesion

NETs induced from neutrophils were incubated with cooled 4% paraformaldehyde (PFA) for 15 min at room temperature. After washing with PBS for three times, the fixed NETs were incubated with DiD‐labeled RM@(PDA/BDP) and DNase‐RM@(PDA/BDP) on NETs for 15 min. After washing with PBS, the NETs were observed with confocal laser scanning microscope (Olympus SpinSR10, Japan) after stained with Hoechst.

### Immunostaining

For cell samples, cells were carefully washed three times after treatment. Then, cells were incubated with cooled 4% paraformaldehyde (PFA) for 15 min at room temperature. After completion, samples were washed three times and incubated with primary antibody anti‐H3cit (1:1000), anti‐MPO (1:1000) and anti‐NE (1:250) at 4 °C overnight. After washing with PBS for three times, the fixed cells were incubated with secondary antibody (1:800) for 2 h at room temperature. The cells were observed with confocal laser scanning microscope (Olympus SpinSR10, Japan) after stained with Hoechst. For brain section, sections were washed three times and incubated with primary antibody anti‐8‐OHG (1:800), anti‐NeuN (1:800), anti‐CD34 (1:50), anti‐GFAP (1:1000), anti‐Ly6G (1:1000), anti‐H3cit (1:1000), anti‐MPO (1:1000), or anti‐NE (1:250) at 4 °C overnight. After washing with PBS for three times, the sections were incubated with secondary antibody (1:800) for 2 h at room temperature. The sections were observed with fluorescence microscope imaging (Olympus VS120, Japan) after stained with DAPI.

### Animals

C57BL/6 mice (male, 18–20 g) were purchased from the Sino‐British SIPPR/BK Lab Animal Ltd. (Shanghai, China). All animal experiments were carried out in accordance with guidelines evaluated and approved by the Fudan University Institutional Animal Care and Use Committee (IACUC). Ethics number: 2018‐03‐YJ‐JC‐02.

### Mouse Transient Middle Cerebral Artery Occlusion/Reperfusion (tMCAO) Model

A reported monofilament method was applied to create artery occlusion. Mouse (20–25 g) were anesthetized with intraperitoneal injection of pentobarbital sodium. The left common carotid artery (CCA), external carotid artery (ECA), and internal carotid artery (ICA) were exposed carefully. The CCA was then briefly ligated with 6‐0 suture, and a small incision was made at the distal end of the ECA. The nylon monofilament was inserted into the ICA through the incision on ECA, the monofilament was advanced until it reached the MCA. After occlusion for 1.5 h, the monofilament was then withdrawn to allow for another 24 h reperfusion. In the sham operation group, the monofilament was immediately withdrawn after reaching the MCA. Then the incision was sutured and mouse was returned to the rearing cage for feeding.

### TTC Staining

The mice were then sacrificed, the removed brains were frozen at −20 °Cand sliced into 1 mm width coronal sections. The brain sections were next stained with 2% TTC in PBS buffer at 37 °C for 30 min, fixed, and imaged with camera. The infarct areas (white part) of brains were quantified with ImageJ software (National Institutes of Health, Maryland, USA).

### Western Blot

Brain tissues were lysed in RIPA buffer containing protease. The lysate solution was centrifuged at 14 000 g for 10 min at 4 °C and the supernatant was collected to obtain the protein. The protein concentrations were quantitated by the Bradford assay. Then, 40 µg of each protein sample was separated on an SDS‐PAGE gel and transferred onto the PVDF membranes (Millipore, St. Louis, USA). After being blocked for 1 h, the membranes were probed with primary antibody anti‐H3cit (1:1000) at 4 °C overnight. Subsequently, horseradish peroxidase (HRP)‐conjugated secondary antibodies were incubated for 1 h. Specific bands were finally visualized using Immobilon Western Chemiluminescent HRP Substrate (Millipore). PVDF membranes were restored and H3 (1:1000) was detected with the same steps.

### BBB Permeability Investigation

Evans blue was intravenously injected into tMCAO model mice. Briefly, 2% Evans blue solution was administrated into tMCAO model mice (4 mL kg^−1^) and brains were obtained after 1 h injection. Fluorescence of Evans blue was detected by IVIS.

### Statistical Analysis

All statistical analyses were performed with GraphPad Prism software (8.0), and all results were reported as means ± standard deviation (SD). The statistical significance between groups was studied by one‐way ANOVA with Tukey post‐test, which was presented as *p < 0.05, **p < 0.01, and ***p < 0.001.

## Conflict of Interest

The authors declare no conflict of interest.

## Supporting information



Supporting Information

## Data Availability

Data sharing is not applicable to this article as no new data were created or analyzed in this study.
