# Supplementary material for: DNase I‐Mediated Chemotactic Nanoparticles for NETs Targeting and Microenvironment Remodeling Treatment of Acute Ischemic Stroke
Source: Adv Sci (Weinh). 2025 Jun 19;12(34):e03689. doi: 10.1002/advs.202503689 (PMC12442667; doi:10.1002/advs.202503689)
Supplement: Supplementary file 1 — Supporting Information [file ADVS-12-e03689-s001.pdf]

ADVANCED SCIENCE
Open Access

Supporting Information

for *Adv. Sci.*, DOI 10.1002/advs.202503689

DNase I-Mediated Chemotactic Nanoparticles for NETs Targeting and Microenvironment Remodeling Treatment of Acute Ischemic Stroke

*Tongyu Zhang, Peixin Liu, Wenru Shen, Chao Li, Zhenhao Zhao, Yuxing Wu, Tao Sun and Chen Jiang**

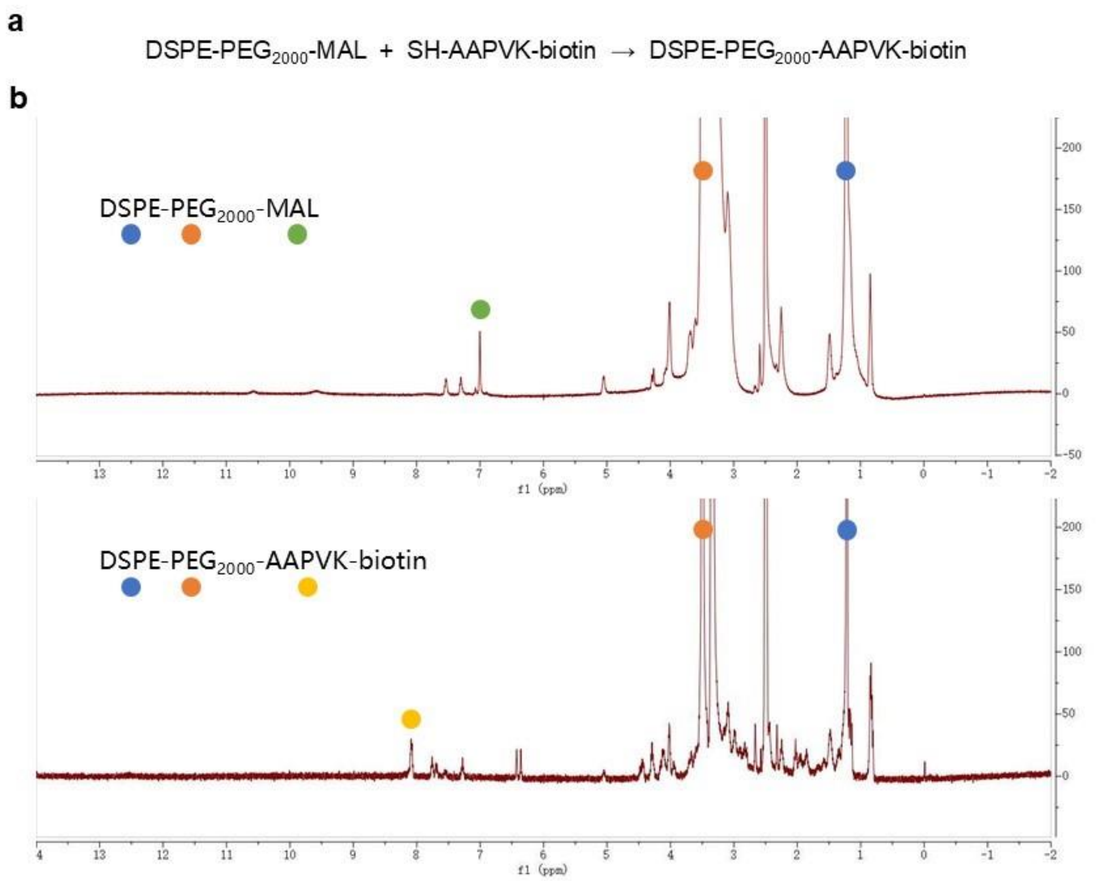

Figure S1. Synthesis and characterization of DSPE-PEG$_{2000}$-AAPVK-biotin. a) Synthesis route. b) ^{1}H-NMR spectrum of DSPE-PEG$_{2000}$-MAL and DSPE-PEG$_{2000}$-AAPVK-biotin.

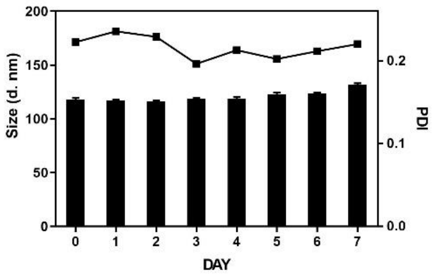

Figure S2. The stability of DNase-RM@(PDA/IDB). Data are presented as mean ± s.d. (n = 3 independent experiments).

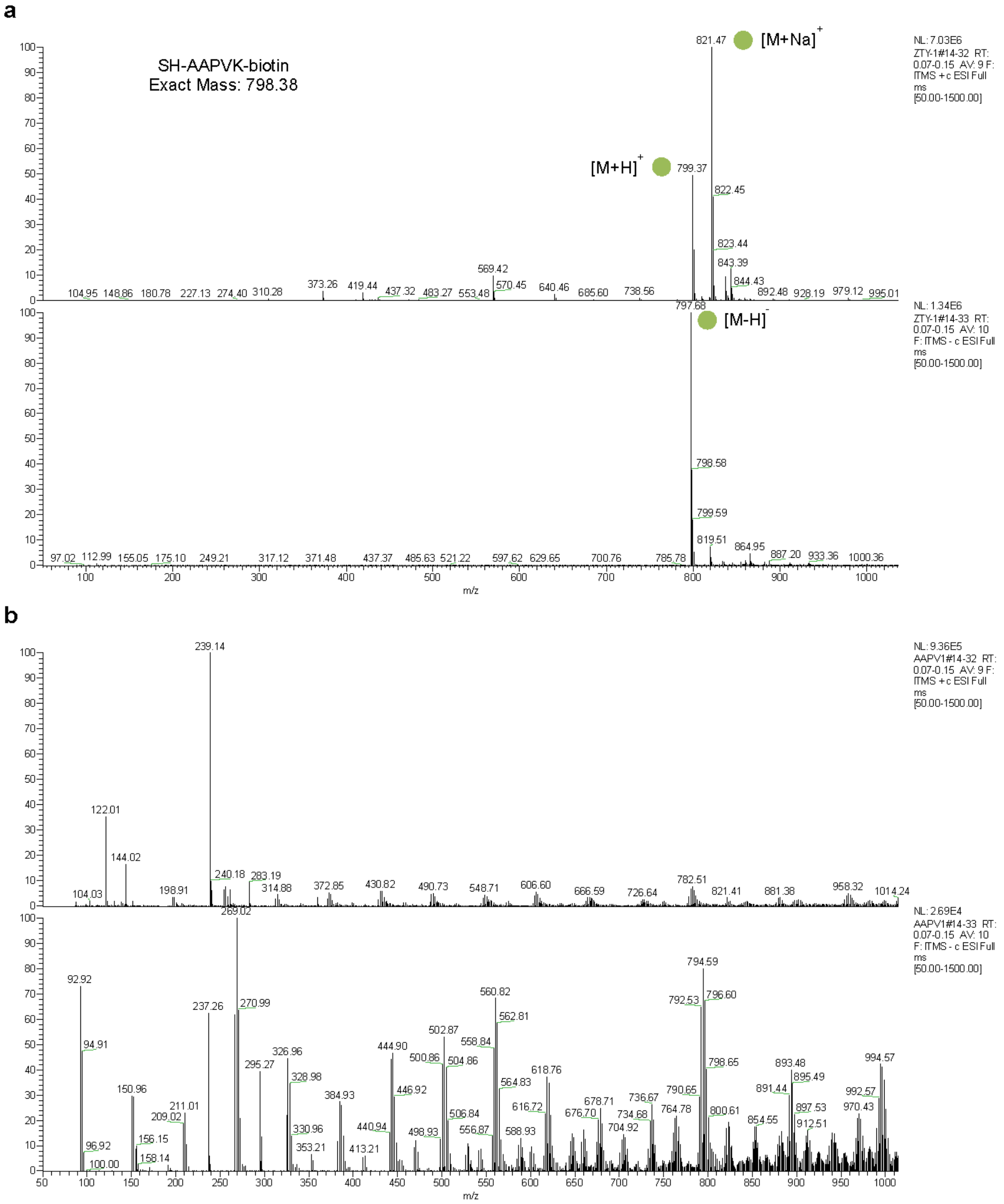

Figure S3. The cleavage of SH-AAPVK-biotin by NE. ESI-MS spectrum of SH-AAPVK-biotin incubated with buffer (a) or NE (b).

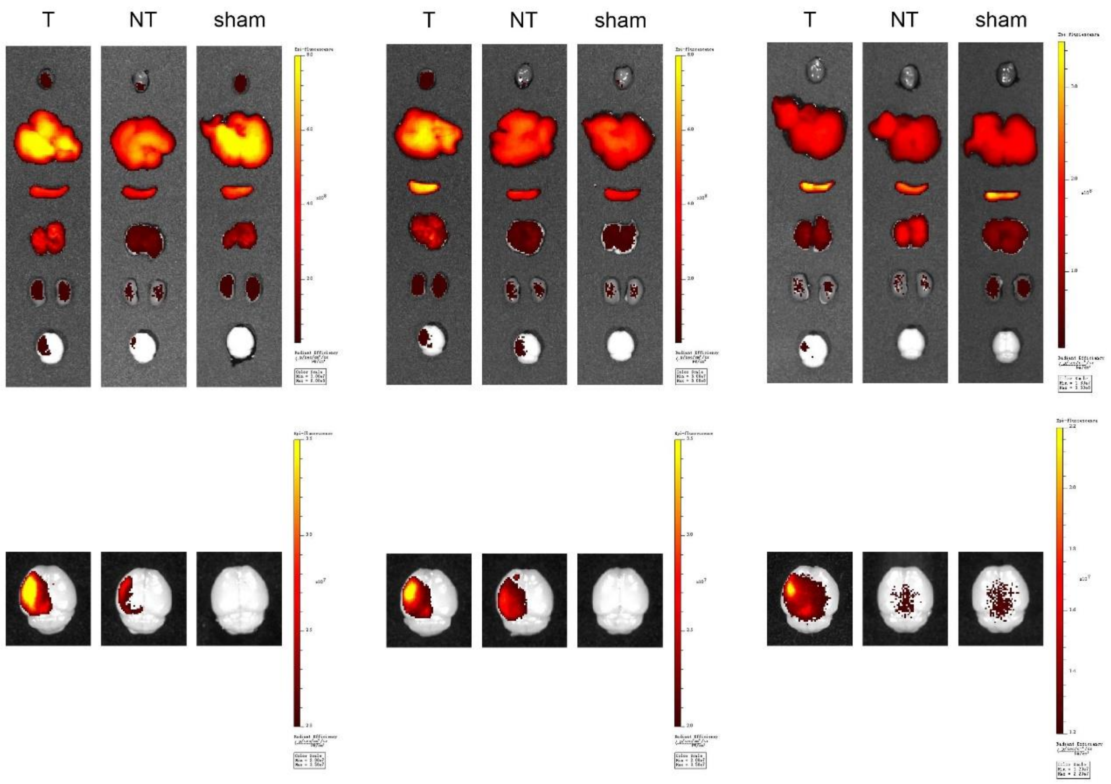

Figure S4. Ex vivo IVIS images of major organs 24 h after intravenous injection.

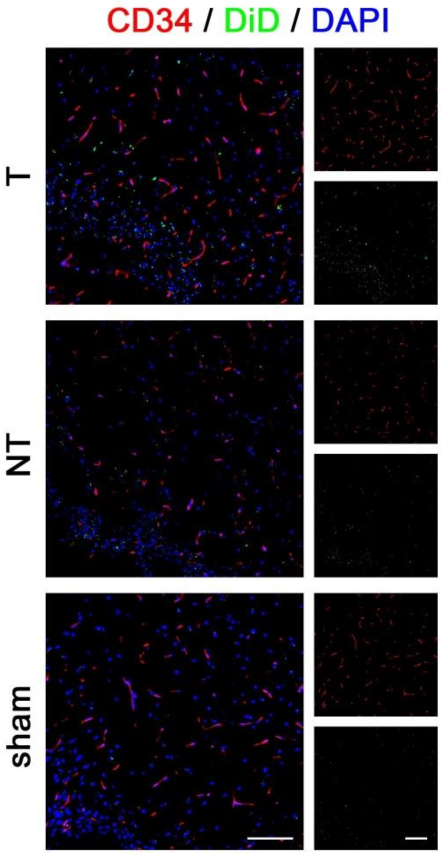

Figure S5. Distribution of DiD and CD34 signals. Scale bars, 100μm.

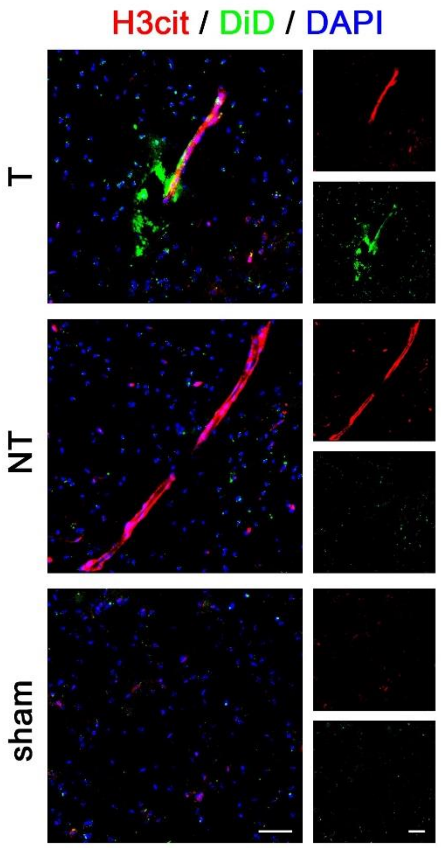

Figure S6. Distribution of DiD and H3cit signals. Scale bars, 100μm.

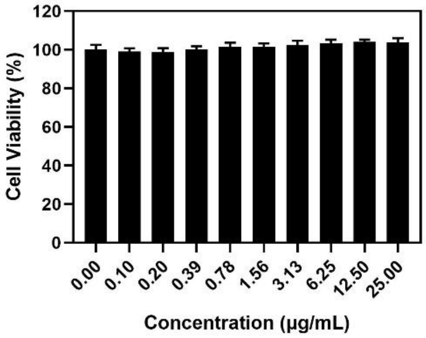

Figure S7. The cytotoxicity of DNase-RM@(PDA/IDB) on SH-SY5Y cells. Data are presented as mean ± s.d. (n = 5 or 6 independent experiments).

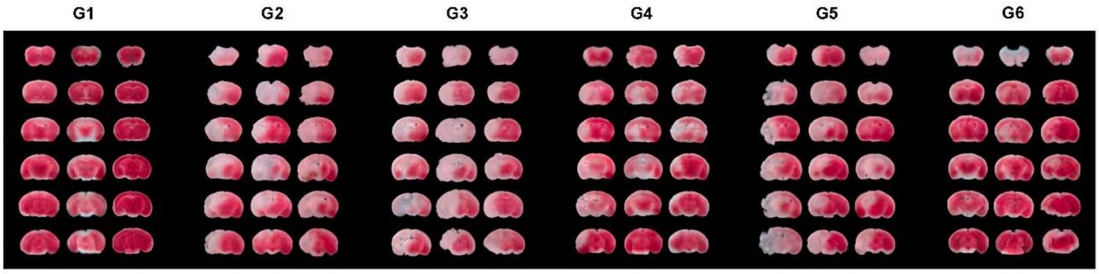

Figure S8. TTC staining images of brain slices.

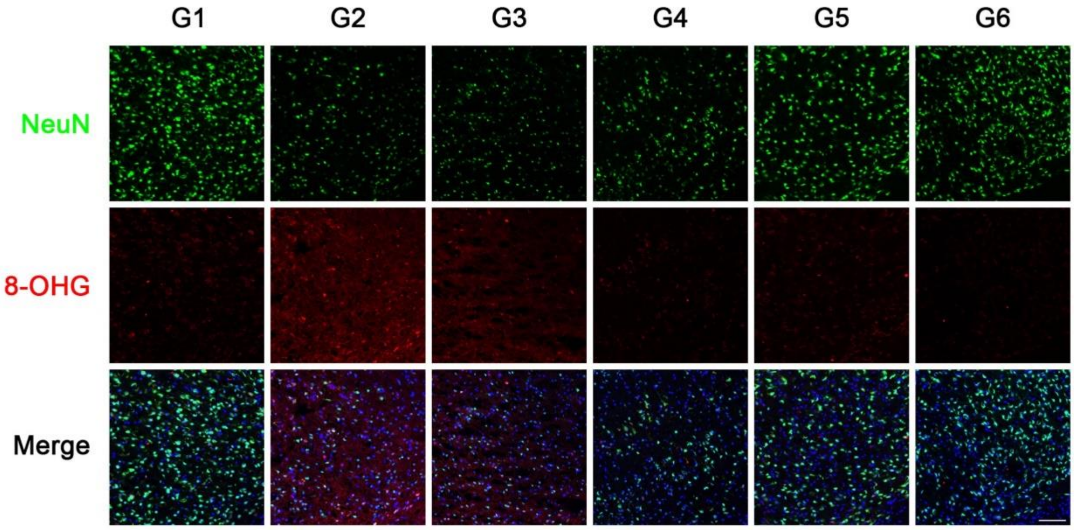

Figure S9. Representative fluorescence images of nerve injury and oxidative stress in MCAO model mice treated with different formulations in the ischemic penumbra. Scale bars, 100 μm.

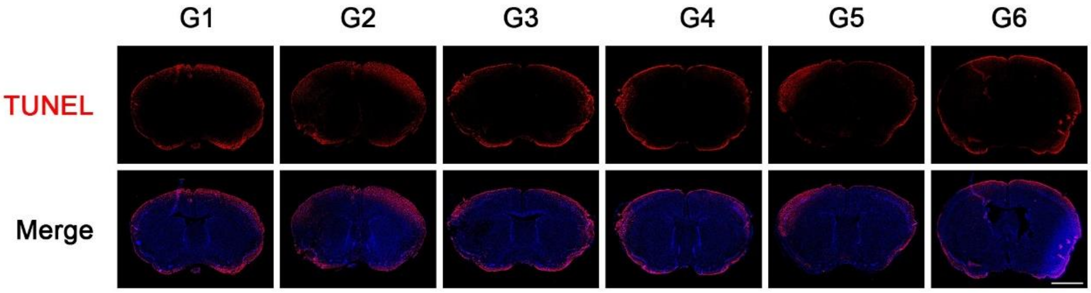

Figure S10. Representative fluorescence images of TUNEL staining. Scale bar, 2 mm.

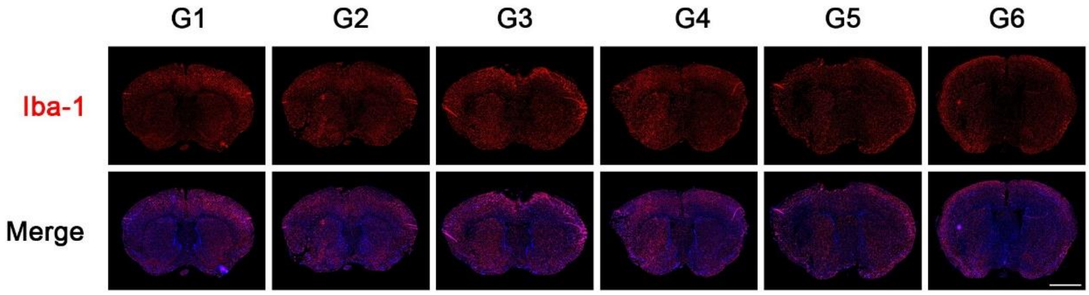

Figure S11. Representative fluorescence images of microglia activation. Scale bars, 2 mm.

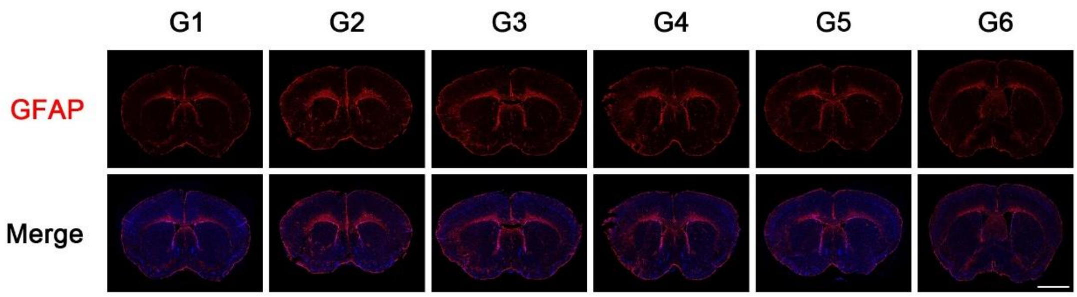

Figure S12. Representative fluorescence images of astrocyte activation. Scale bars, 2 mm.

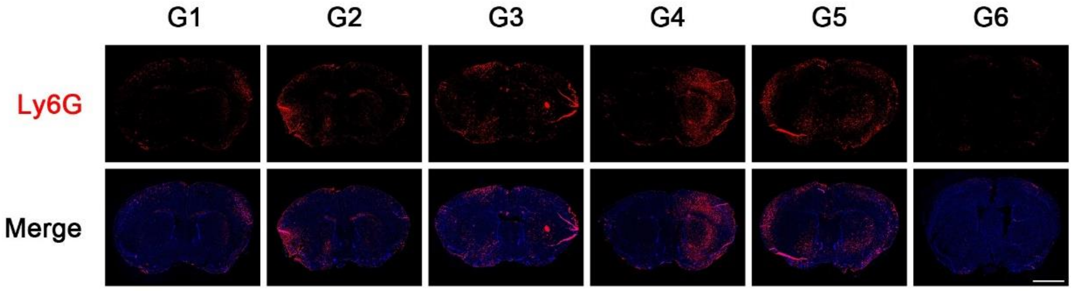

Figure S13. Representative fluorescence images of neutrophil infiltration. Scale bar, 2 mm.

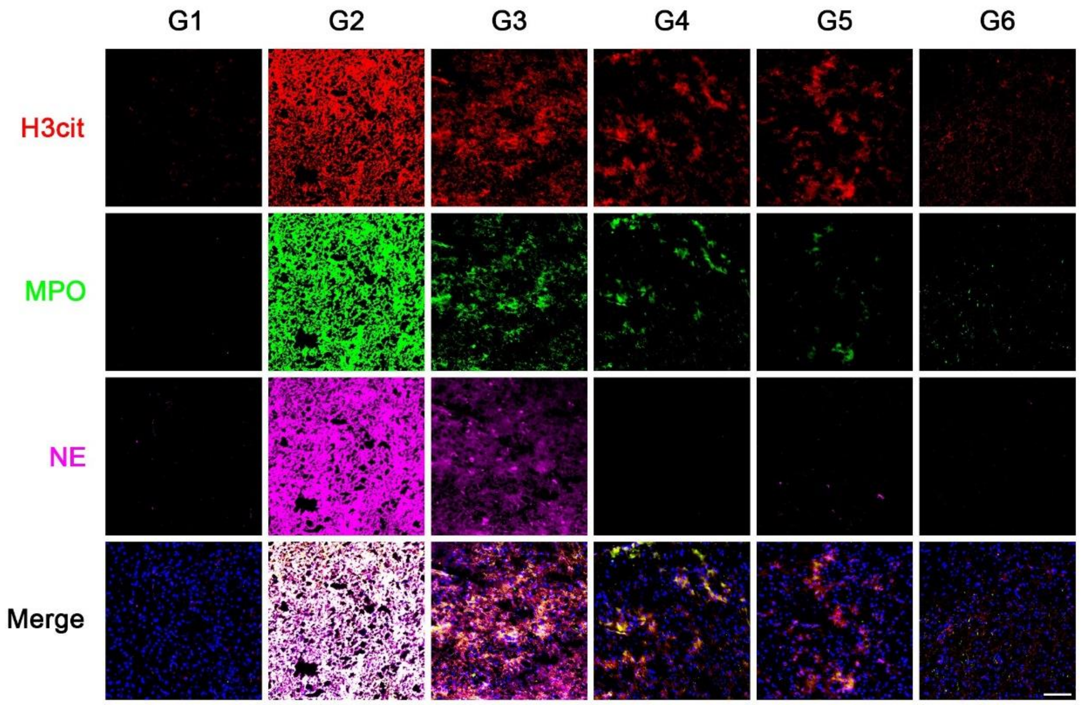

Figure S14. Representative fluorescence images of NETs markers in the ischemic penumbra. Scale bars, 100 μm.

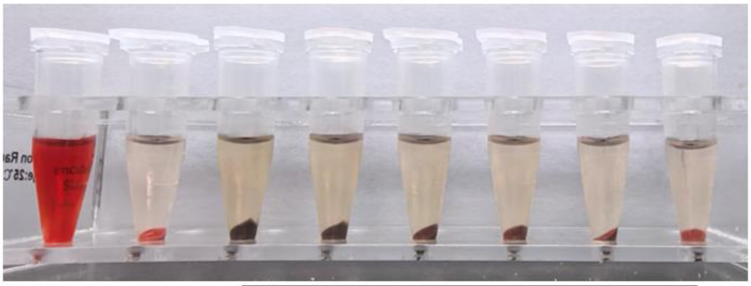

Figure S15. Investigation of hemolysis. Photograph of the centrifuged samples used for the hemolysis assay.

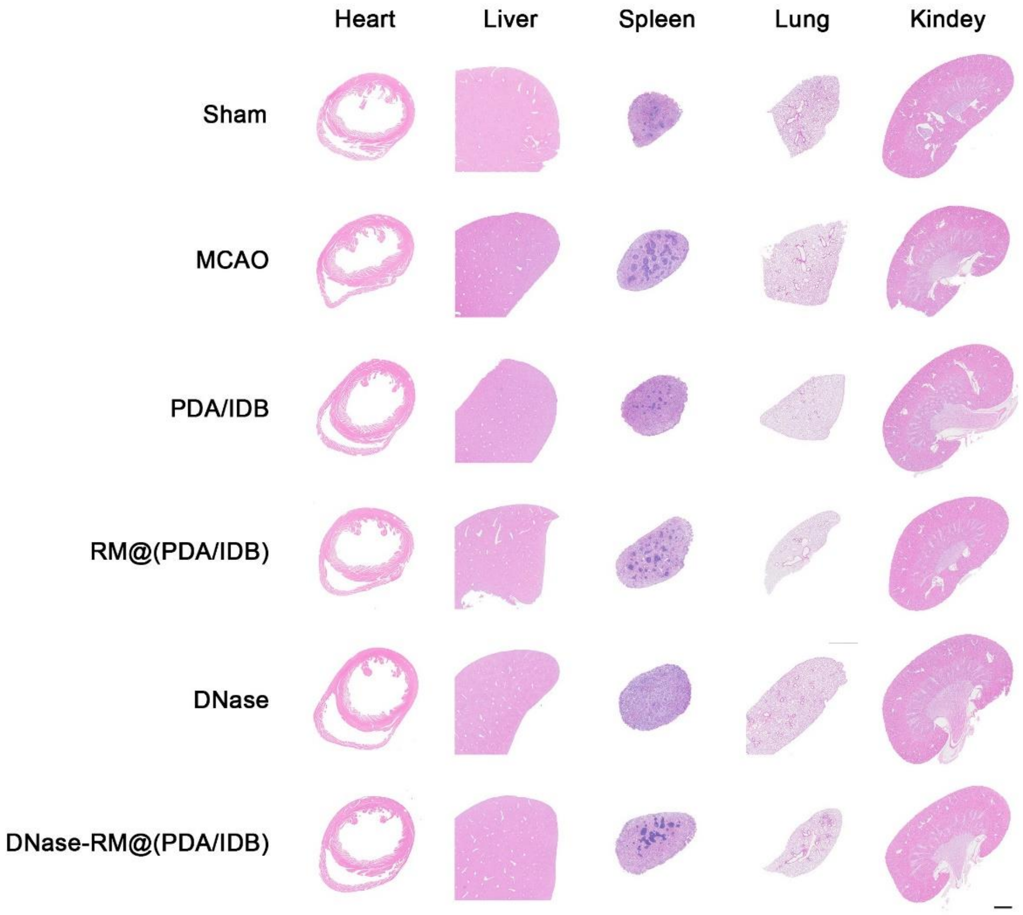

Figure S16. Representative histochemistry analysis of heart, liver, spleen, lung, and kidney sections from different mice stained with HE. Scale bars, 1 mm.

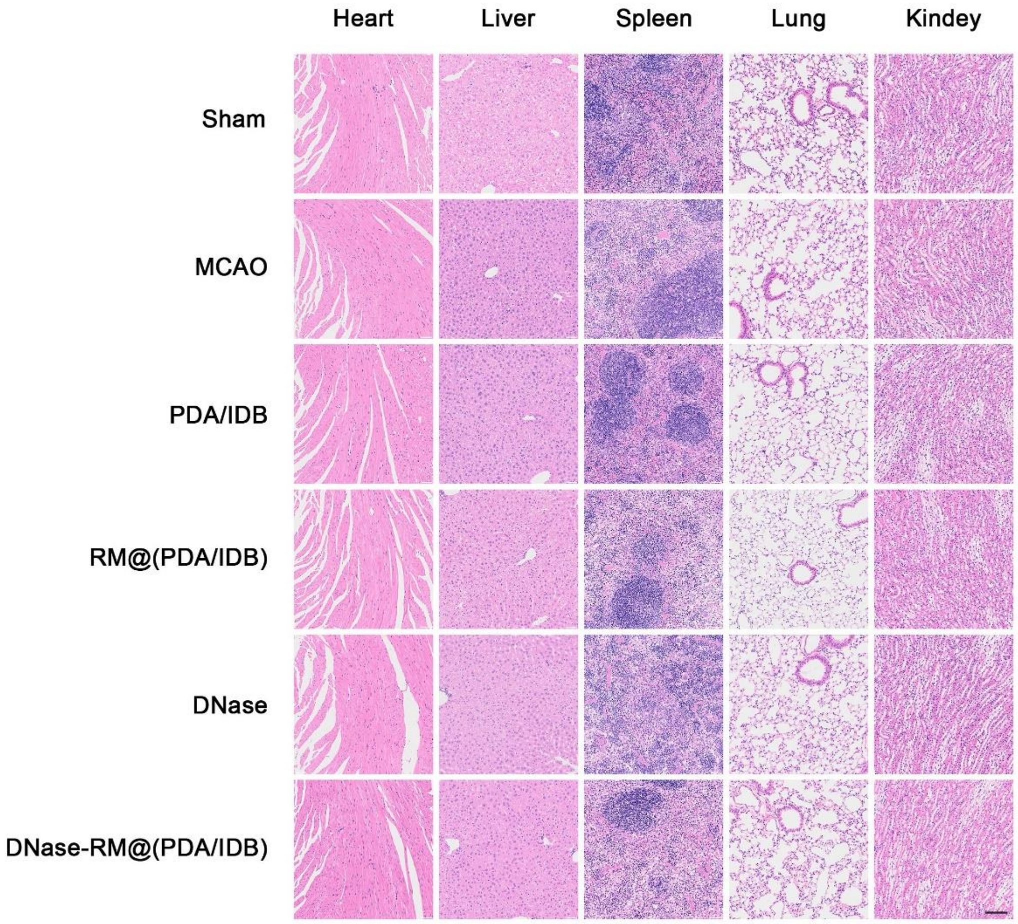

Figure S17. Representative histochemistry analysis of heart, liver, spleen, lung, and kidney sections from different mice stained with HE. Scale bars, 100 μm.

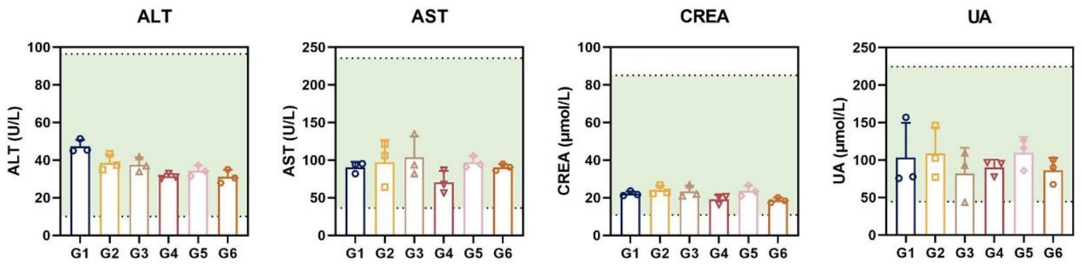

Figure S18. ALT, AST, CREA and UA levels in the plasma from mice 7 days after different treatments. Data are presented as mean ± s.d. (n = 3 independent experiments).
